# 6,6′-Dimeth­oxy-2,2′,3,3′,5-penta­nitro-1,1′-biphen­yl

**DOI:** 10.1107/S1600536808011926

**Published:** 2008-05-03

**Authors:** Yuan-Yuan Jiang, Shao-Bin Miao, Dong-Sheng Deng, Bao-Ming Ji

**Affiliations:** a College of Chemistry and Molecular Engineering, Qingdao University of Science and Technology, Qingdao 266042, People’s Republic of China; b College of Chemistry and Chemical Engineering, Luoyang Normal University, Luoyang 471022, People’s Republic of China

## Abstract

In the axially  chiral title compound, C_14_H_9_N_5_O_12_, the dihedral angle between the two benzene rings is 86.0 (8)°. In the crystal structure, the mol­ecules display a two-dimensional framework formed by weak inter­molecular C—H⋯O hydrogen bonds.

## Related literature

For related literature, see: Chen *et al.* (2001 ▶); Fischer *et al.* (2007 ▶); Narayanan *et al.* (2005 ▶); Saito & Koizumi (2005 ▶); Xiao *et al.* (2007 ▶); Yang *et al.* (2005 ▶).
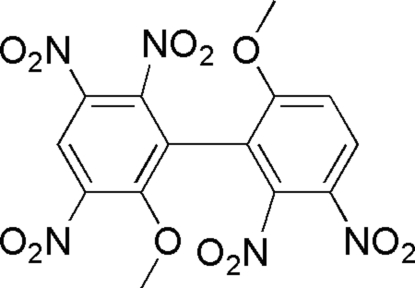


## Experimental

### 

#### Crystal data

C_14_H_9_N_5_O_12_
*M* *_r_* = 439.26
Triclinic, 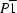

*a* = 10.3765 (13) Å
*b* = 10.4423 (13) Å
*c* = 10.4429 (13) Å
α = 82.5650 (10)°
β = 62.2850 (10)°
γ = 60.5200 (10)°
*V* = 864.73 (19) Å^3^
*Z* = 2
Mo *K*α radiation
μ = 0.15 mm^−1^
*T* = 291 (2) K
0.41 × 0.34 × 0.29 mm

#### Data collection

Bruker APEXII CCD area-detector diffractometer
Absorption correction: multi-scan (*SADABS*; Sheldrick, 1996 ▶) *T*_min_ = 0.940, *T*_max_ = 0.958
6598 measured reflections
3194 independent reflections
2686 reflections with *I* > 2σ(*I*)
*R*_int_ = 0.013

#### Refinement

*R*[*F* ^2^ > 2σ(*F* ^2^)] = 0.041
*wR*(*F* ^2^) = 0.117
*S* = 1.02
3194 reflections
282 parameters
H-atom parameters constrained
Δρ_max_ = 0.25 e Å^−3^
Δρ_min_ = −0.21 e Å^−3^

### 

Data collection: *APEX2* (Bruker, 2004 ▶); cell refinement: *SAINT* (Bruker, 2004 ▶); data reduction: *SAINT*; program(s) used to solve structure: *SHELXS97* (Sheldrick, 2008 ▶); program(s) used to refine structure: *SHELXL97* (Sheldrick, 2008 ▶); molecular graphics: *SHELXTL* (Sheldrick, 2008 ▶); software used to prepare material for publication: *SHELXTL*.

## Supplementary Material

Crystal structure: contains datablocks global, I. DOI: 10.1107/S1600536808011926/si2086sup1.cif

Structure factors: contains datablocks I. DOI: 10.1107/S1600536808011926/si2086Isup2.hkl

Additional supplementary materials:  crystallographic information; 3D view; checkCIF report

## Figures and Tables

**Table 1:** Hydrogen-bond geometry (Å, °)

*D*—H⋯*A*	*D*—H	H⋯*A*	*D*⋯*A*	*D*—H⋯*A*
C13—H13*A*⋯O3	0.96	2.45	2.926 (3)	111
C14—H14*B*⋯O10^i^	0.96	2.55	3.502 (3)	174
C14—H14*C*⋯O8^ii^	0.96	2.58	3.371 (3)	140

Symmetry codes: (i) 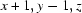; (ii) 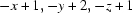.

**Fig. 1.**
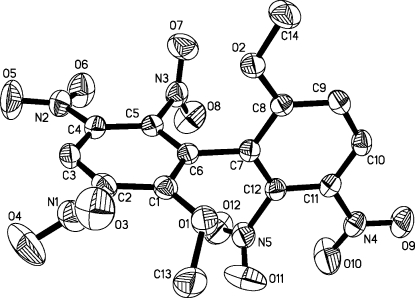
Atom numbering scheme for the title compound with 30% probability displacement ellipsoids.

**Fig. 2.**
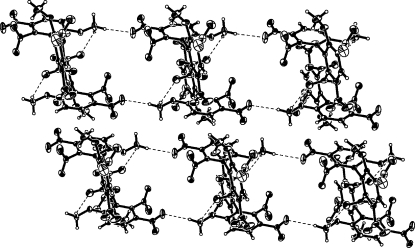
View of the two-dimensional sheet structure. (C—H···O interactions are represented as broken lines).

## supplementary crystallographic information

### Comment

Nitro compounds, specially aromatic nitro compounds have been widely studied owing to their potential application in, for example, pathology (Narayanan, *et al.*, 2005), materials science (Saito & Koizumi, 2005). On the other hand, in our search for chiral compounds, the title related chiral 6,6'-dimethoxy-2,3,2',5'-tetranitro-1,1'-biphenyl compound was synthesized by Xiao *et al.*, (2007). Herein, as an extension to our previous investigation, we report the synthesis and structural characterization of the title compound.

In contrast to our highly substituted biphenyl compounds, the unsubstituted biphenyl groups in compounds synthesized by Fischer *et al.*, (2007) were found to be approximately planar. The molecular geometry in the title compound displays special behavior, the dihedral angle between the benzene rings is 94.0 (8)°, and all the nitro groups at positions 2,3,5,2',3' are twisted out of the corresponding rings which is 45.5 (3)°, 13.5 (5)°, 98.4 (3)°, 6.6 (4)° and 83.5 (5)°, respectively, as depicted in Fig.1. Bond lengths and angles are in good agreement with the dinitrophenyl group in the structure of 1-(2,4-dinitrophenyl)azo-1-nitrocyclohexane, reported by Yang *et al.*, (2005). One intramolecular C—H···O hydrogen bond is observed in the title molecule, and the two intermolecular C—H···O hydrogen bonding contacts (Table 1) form closed two-dimensional grid motifs (Fig. 2).

### Experimental

All chemicals and solvents purchased were of reagent grade and used without further purification. The precursor 6,6'-Dimethoxy-2,2'-dinitro-1,1'-biphenyl was prepared according to the reported procedure (Chen *et al.*, 2001). However, the title compound was obtained by chance when we tried to prepare the 6,6'-Dimethoxy-2,3,5,2',3',5'-hexanitro-1,1'-biphenyl compound. That is, the title compound was synthesized by the nitration reaction of the precursor (0.5 mmol) in 10 ml of concentrated nitric acid at room temperature for 24 h. The resulting solution was poured into 30 ml of ice water and the resulting precipitate was collected by filtration and recrystallized from ethyl acetate to obtain the title crystals, which were suitable for X-ray diffraction analysis. Cautious, the title compound has potential explosive property.

### Refinement

H atoms were positioned geometrically and treated as riding, with C—H bonding lengths constrained to 0.93 (aromatic H), 0.96 Å (methyl H), and with *U*ĩso~(H) = 1.2Ueq (aromatic H) or *U*ĩso~(H) = 1.5Ueq (methyl H).

### Crystal data

**Table:**

C_14_H_9_N_5_O_12_	*Z* = 2
*M**_r_* = 439.26	*F*_000_ = 448
Triclinic, *P*1	*D*_x_ = 1.687 Mg m^−^^3^
Hall symbol: -P 1	Mo *K*α radiation λ = 0.71073 Å
*a* = 10.3765 (13) Å	Cell parameters from 2971 reflections
*b* = 10.4423 (13) Å	θ = 2.4–25.5º
*c* = 10.4429 (13) Å	µ = 0.15 mm^−^^1^
α = 82.5650 (10)º	*T* = 291 (2) K
β = 62.2850 (10)º	Block, yellow
γ = 60.5200 (10)º	0.41 × 0.34 × 0.29 mm
*V* = 864.73 (19) Å^3^	

### Data collection

**Table:**

Bruker APEXII CCD area-detector diffractometer	3194 independent reflections
Radiation source: fine-focus sealed tube	2686 reflections with *I* > 2σ(*I*)
Monochromator: graphite	*R*_int_ = 0.013
*T* = 291(2) K	θ_max_ = 25.5º
φ and ω scans	θ_min_ = 2.4º
Absorption correction: multi-scan(SADABS; Sheldrick, 1996)	*h* = −12→12
*T*_min_ = 0.940, *T*_max_ = 0.958	*k* = −12→12
6598 measured reflections	*l* = −12→12

### Refinement

**Table:**

Refinement on *F*^2^	Secondary atom site location: difference Fourier map
Least-squares matrix: full	Hydrogen site location: inferred from neighbouring sites
*R*[*F*^2^ > 2σ(*F*^2^)] = 0.041	H-atom parameters constrained
*wR*(*F*^2^) = 0.117	*w* = 1/[σ^2^(*F*_o_^2^) + (0.059*P*)^2^ + 0.3851*P*] where *P* = (*F*_o_^2^ + 2*F*_c_^2^)/3
*S* = 1.02	(Δ/σ)_max_ < 0.001
3194 reflections	Δρ_max_ = 0.25 e Å^−^^3^
282 parameters	Δρ_min_ = −0.21 e Å^−^^3^
Primary atom site location: structure-invariant direct methods	Extinction correction: none

### Special details

**Table:**

Geometry. All e.s.d.'s (except the e.s.d. in the dihedral angle between two l.s. planes) are estimated using the full covariance matrix. The cell e.s.d.'s are taken into account individually in the estimation of e.s.d.'s in distances, angles and torsion angles; correlations between e.s.d.'s in cell parameters are only used when they are defined by crystal symmetry. An approximate (isotropic) treatment of cell e.s.d.'s is used for estimating e.s.d.'s involving l.s. planes.
Refinement. Refinement of *F*^2^ against ALL reflections. The weighted *R*-factor *wR* and goodness of fit *S* are based on *F*^2^, conventional *R*-factors *R* are based on *F*, with *F* set to zero for negative *F*^2^. The threshold expression of *F*^2^ > σ(*F*^2^) is used only for calculating *R*-factors(gt) *etc*. and is not relevant to the choice of reflections for refinement. *R*-factors based on *F*^2^ are statistically about twice as large as those based on *F*, and *R*- factors based on ALL data will be even larger.

### Fractional atomic coordinates and isotropic or equivalent isotropic displacement parameters (Å^2^)

**Table:**

	*x*	*y*	*z*	*U*_iso_*/*U*_eq_	
O1	0.36354 (18)	0.70656 (16)	0.19497 (14)	0.0448 (3)	
O2	0.69388 (15)	0.72440 (14)	0.24022 (14)	0.0382 (3)	
O3	0.4217 (2)	0.40331 (19)	0.2500 (2)	0.0678 (5)	
O4	0.1905 (2)	0.4489 (2)	0.4428 (3)	0.0852 (6)	
O5	0.2186 (3)	0.6187 (2)	0.84423 (19)	0.0803 (6)	
O6	0.2172 (2)	0.8273 (2)	0.83302 (16)	0.0648 (5)	
O7	0.5003 (2)	0.8533 (2)	0.57692 (17)	0.0582 (4)	
O8	0.2549 (2)	1.03379 (17)	0.61824 (19)	0.0640 (5)	
O9	0.2989 (2)	1.33902 (17)	0.06894 (18)	0.0571 (4)	
O10	0.0962 (2)	1.3106 (2)	0.2303 (2)	0.0846 (7)	
O11	0.0882 (2)	1.0384 (2)	0.2635 (2)	0.0761 (6)	
O12	0.04620 (19)	1.1239 (2)	0.46358 (18)	0.0702 (5)	
N1	0.3036 (2)	0.4748 (2)	0.3675 (2)	0.0507 (5)	
N2	0.2378 (2)	0.7181 (2)	0.77767 (18)	0.0484 (4)	
N3	0.3645 (2)	0.90493 (19)	0.58132 (16)	0.0404 (4)	
N4	0.2444 (2)	1.26902 (17)	0.15879 (17)	0.0391 (4)	
N5	0.1327 (2)	1.06936 (18)	0.3386 (2)	0.0433 (4)	
C1	0.3442 (2)	0.69672 (19)	0.33107 (19)	0.0316 (4)	
C2	0.3056 (2)	0.59612 (19)	0.4225 (2)	0.0351 (4)	
C3	0.2736 (2)	0.6019 (2)	0.5661 (2)	0.0371 (4)	
H3	0.2441	0.5362	0.6249	0.045*	
C4	0.2859 (2)	0.7060 (2)	0.62141 (19)	0.0351 (4)	
C5	0.3359 (2)	0.80022 (19)	0.52985 (19)	0.0314 (4)	
C6	0.3661 (2)	0.79704 (18)	0.38642 (18)	0.0283 (4)	
C7	0.4211 (2)	0.89880 (18)	0.28967 (17)	0.0280 (4)	
C8	0.5928 (2)	0.85656 (18)	0.21610 (18)	0.0287 (4)	
C9	0.6454 (2)	0.9505 (2)	0.12600 (18)	0.0330 (4)	
H9	0.7584	0.9224	0.0769	0.040*	
C10	0.5303 (2)	1.08464 (19)	0.10979 (18)	0.0331 (4)	
H10	0.5660	1.1471	0.0507	0.040*	
C11	0.3623 (2)	1.12714 (18)	0.18047 (18)	0.0308 (4)	
C12	0.3096 (2)	1.03275 (19)	0.26904 (18)	0.0302 (4)	
C13	0.2548 (4)	0.6888 (3)	0.1570 (3)	0.0651 (7)	
H13A	0.3124	0.5908	0.1070	0.098*	
H13B	0.2243	0.7609	0.0946	0.098*	
H13C	0.1559	0.7028	0.2442	0.098*	
C14	0.8715 (2)	0.6715 (2)	0.1654 (3)	0.0536 (6)	
H14A	0.9102	0.6645	0.0619	0.080*	
H14B	0.9285	0.5755	0.1910	0.080*	
H14C	0.8939	0.7394	0.1934	0.080*	

### Atomic displacement parameters (Å^2^)

**Table:**

	*U*^11^	*U*^22^	*U*^33^	*U*^12^	*U*^13^	*U*^23^
O1	0.0591 (9)	0.0572 (9)	0.0359 (7)	−0.0397 (8)	−0.0238 (7)	0.0089 (6)
O2	0.0289 (6)	0.0334 (7)	0.0451 (7)	−0.0130 (5)	−0.0157 (6)	0.0099 (5)
O3	0.0824 (13)	0.0479 (9)	0.0718 (12)	−0.0313 (9)	−0.0322 (10)	−0.0061 (8)
O4	0.0745 (13)	0.0756 (13)	0.1234 (17)	−0.0582 (11)	−0.0347 (12)	0.0068 (12)
O5	0.1272 (17)	0.1048 (15)	0.0559 (10)	−0.0899 (15)	−0.0508 (11)	0.0482 (10)
O6	0.0882 (13)	0.0741 (12)	0.0384 (8)	−0.0475 (10)	−0.0264 (8)	0.0091 (8)
O7	0.0600 (10)	0.0829 (12)	0.0526 (9)	−0.0446 (9)	−0.0309 (8)	0.0070 (8)
O8	0.0900 (13)	0.0396 (9)	0.0696 (11)	−0.0276 (9)	−0.0460 (10)	0.0029 (7)
O9	0.0681 (10)	0.0444 (8)	0.0624 (10)	−0.0317 (8)	−0.0342 (8)	0.0286 (7)
O10	0.0399 (9)	0.0592 (11)	0.1113 (16)	−0.0129 (8)	−0.0246 (10)	0.0458 (11)
O11	0.0482 (10)	0.0769 (12)	0.1132 (16)	−0.0263 (9)	−0.0453 (10)	−0.0055 (11)
O12	0.0383 (8)	0.0812 (12)	0.0476 (10)	−0.0124 (8)	−0.0077 (7)	0.0123 (9)
N1	0.0541 (11)	0.0396 (9)	0.0703 (13)	−0.0277 (9)	−0.0332 (10)	0.0094 (9)
N2	0.0543 (11)	0.0646 (12)	0.0385 (9)	−0.0381 (10)	−0.0243 (8)	0.0213 (9)
N3	0.0540 (10)	0.0481 (10)	0.0313 (8)	−0.0321 (9)	−0.0224 (7)	0.0098 (7)
N4	0.0467 (10)	0.0305 (8)	0.0402 (9)	−0.0181 (7)	−0.0226 (8)	0.0103 (7)
N5	0.0327 (8)	0.0344 (9)	0.0536 (11)	−0.0137 (7)	−0.0189 (8)	0.0149 (7)
C1	0.0280 (8)	0.0314 (9)	0.0333 (9)	−0.0139 (7)	−0.0132 (7)	0.0033 (7)
C2	0.0309 (9)	0.0296 (9)	0.0464 (10)	−0.0163 (7)	−0.0173 (8)	0.0042 (8)
C3	0.0335 (9)	0.0347 (10)	0.0450 (11)	−0.0203 (8)	−0.0185 (8)	0.0158 (8)
C4	0.0338 (9)	0.0400 (10)	0.0328 (9)	−0.0198 (8)	−0.0162 (8)	0.0116 (8)
C5	0.0305 (9)	0.0312 (9)	0.0340 (9)	−0.0153 (7)	−0.0166 (7)	0.0064 (7)
C6	0.0247 (8)	0.0257 (8)	0.0322 (9)	−0.0112 (7)	−0.0132 (7)	0.0055 (7)
C7	0.0317 (9)	0.0279 (8)	0.0263 (8)	−0.0158 (7)	−0.0136 (7)	0.0036 (6)
C8	0.0308 (9)	0.0288 (8)	0.0281 (8)	−0.0143 (7)	−0.0147 (7)	0.0022 (7)
C9	0.0314 (9)	0.0375 (9)	0.0306 (9)	−0.0202 (8)	−0.0109 (7)	0.0026 (7)
C10	0.0427 (10)	0.0336 (9)	0.0288 (9)	−0.0250 (8)	−0.0146 (8)	0.0060 (7)
C11	0.0383 (9)	0.0271 (8)	0.0288 (8)	−0.0156 (7)	−0.0177 (7)	0.0048 (7)
C12	0.0303 (9)	0.0311 (9)	0.0281 (8)	−0.0153 (7)	−0.0127 (7)	0.0037 (7)
C13	0.098 (2)	0.0792 (18)	0.0680 (15)	−0.0620 (16)	−0.0593 (15)	0.0260 (13)
C14	0.0295 (10)	0.0445 (12)	0.0705 (15)	−0.0120 (9)	−0.0182 (10)	0.0085 (10)

### Geometric parameters (Å, °)

**Table:**

O1—C1	1.337 (2)	C2—C3	1.380 (3)
O1—C13	1.451 (3)	C3—C4	1.379 (3)
O2—C8	1.341 (2)	C3—H3	0.9300
O2—C14	1.443 (2)	C4—C5	1.396 (2)
O3—N1	1.225 (3)	C5—C6	1.382 (2)
O4—N1	1.211 (2)	C6—C7	1.503 (2)
O5—N2	1.220 (2)	C7—C12	1.379 (2)
O6—N2	1.223 (2)	C7—C8	1.414 (2)
O7—N3	1.215 (2)	C8—C9	1.399 (2)
O8—N3	1.214 (2)	C9—C10	1.377 (3)
O9—N4	1.212 (2)	C9—H9	0.9300
O10—N4	1.212 (2)	C10—C11	1.381 (3)
O11—N5	1.218 (2)	C10—H10	0.9300
O12—N5	1.197 (2)	C11—C12	1.396 (2)
N1—C2	1.470 (2)	C13—H13A	0.9600
N2—C4	1.470 (2)	C13—H13B	0.9600
N3—C5	1.481 (2)	C13—H13C	0.9600
N4—C11	1.459 (2)	C14—H14A	0.9600
N5—C12	1.478 (2)	C14—H14B	0.9600
C1—C2	1.405 (2)	C14—H14C	0.9600
C1—C6	1.413 (2)		
			
C1—O1—C13	120.36 (16)	C5—C6—C7	120.87 (15)
C8—O2—C14	118.30 (14)	C1—C6—C7	119.98 (15)
O4—N1—O3	124.87 (19)	C12—C7—C8	118.36 (15)
O4—N1—C2	118.4 (2)	C12—C7—C6	122.25 (15)
O3—N1—C2	116.66 (17)	C8—C7—C6	119.39 (14)
O5—N2—O6	124.23 (18)	O2—C8—C9	125.08 (15)
O5—N2—C4	117.39 (18)	O2—C8—C7	115.04 (14)
O6—N2—C4	118.38 (16)	C9—C8—C7	119.88 (15)
O8—N3—O7	126.09 (18)	C10—C9—C8	120.20 (16)
O8—N3—C5	117.95 (16)	C10—C9—H9	119.9
O7—N3—C5	115.89 (17)	C8—C9—H9	119.9
O9—N4—O10	122.95 (17)	C9—C10—C11	120.58 (16)
O9—N4—C11	118.70 (16)	C9—C10—H10	119.7
O10—N4—C11	118.34 (15)	C11—C10—H10	119.7
O12—N5—O11	125.41 (19)	C10—C11—C12	119.36 (16)
O12—N5—C12	118.04 (18)	C10—C11—N4	118.98 (15)
O11—N5—C12	116.54 (17)	C12—C11—N4	121.66 (16)
O1—C1—C2	126.17 (16)	C7—C12—C11	121.61 (16)
O1—C1—C6	116.23 (15)	C7—C12—N5	117.27 (15)
C2—C1—C6	117.61 (16)	C11—C12—N5	121.06 (15)
C3—C2—C1	122.38 (16)	O1—C13—H13A	109.5
C3—C2—N1	116.34 (16)	O1—C13—H13B	109.5
C1—C2—N1	121.24 (17)	H13A—C13—H13B	109.5
C2—C3—C4	119.28 (16)	O1—C13—H13C	109.5
C2—C3—H3	120.4	H13A—C13—H13C	109.5
C4—C3—H3	120.4	H13B—C13—H13C	109.5
C3—C4—C5	119.40 (16)	O2—C14—H14A	109.5
C3—C4—N2	118.23 (16)	O2—C14—H14B	109.5
C5—C4—N2	122.31 (17)	H14A—C14—H14B	109.5
C6—C5—C4	121.87 (16)	O2—C14—H14C	109.5
C6—C5—N3	116.95 (15)	H14A—C14—H14C	109.5
C4—C5—N3	121.15 (15)	H14B—C14—H14C	109.5
C5—C6—C1	119.15 (15)		
			
C13—O1—C1—C2	39.2 (3)	C2—C1—C6—C7	175.07 (15)
C13—O1—C1—C6	−141.22 (19)	C5—C6—C7—C12	−95.4 (2)
O1—C1—C2—C3	−174.24 (17)	C1—C6—C7—C12	84.4 (2)
C6—C1—C2—C3	6.2 (3)	C5—C6—C7—C8	84.9 (2)
O1—C1—C2—N1	8.1 (3)	C1—C6—C7—C8	−95.26 (19)
C6—C1—C2—N1	−171.47 (16)	C14—O2—C8—C9	−1.7 (3)
O4—N1—C2—C3	45.1 (3)	C14—O2—C8—C7	178.11 (16)
O3—N1—C2—C3	−132.0 (2)	C12—C7—C8—O2	−179.37 (14)
O4—N1—C2—C1	−137.1 (2)	C6—C7—C8—O2	0.3 (2)
O3—N1—C2—C1	45.8 (3)	C12—C7—C8—C9	0.4 (2)
C1—C2—C3—C4	−2.5 (3)	C6—C7—C8—C9	−179.88 (15)
N1—C2—C3—C4	175.21 (16)	O2—C8—C9—C10	−179.60 (15)
C2—C3—C4—C5	−2.2 (3)	C7—C8—C9—C10	0.6 (2)
C2—C3—C4—N2	175.03 (16)	C8—C9—C10—C11	−0.9 (3)
O5—N2—C4—C3	12.9 (3)	C9—C10—C11—C12	0.0 (2)
O6—N2—C4—C3	−166.04 (19)	C9—C10—C11—N4	−179.06 (15)
O5—N2—C4—C5	−170.01 (19)	O9—N4—C11—C10	6.8 (2)
O6—N2—C4—C5	11.1 (3)	O10—N4—C11—C10	−174.16 (19)
C3—C4—C5—C6	3.1 (3)	O9—N4—C11—C12	−172.27 (17)
N2—C4—C5—C6	−173.96 (16)	O10—N4—C11—C12	6.8 (3)
C3—C4—C5—N3	−174.78 (16)	C8—C7—C12—C11	−1.3 (2)
N2—C4—C5—N3	8.2 (3)	C6—C7—C12—C11	179.06 (15)
O8—N3—C5—C6	81.7 (2)	C8—C7—C12—N5	175.72 (15)
O7—N3—C5—C6	−95.50 (19)	C6—C7—C12—N5	−3.9 (2)
O8—N3—C5—C4	−100.3 (2)	C10—C11—C12—C7	1.0 (2)
O7—N3—C5—C4	82.5 (2)	N4—C11—C12—C7	−179.88 (15)
C4—C5—C6—C1	0.6 (3)	C10—C11—C12—N5	−175.84 (16)
N3—C5—C6—C1	178.61 (15)	N4—C11—C12—N5	3.2 (2)
C4—C5—C6—C7	−179.53 (15)	O12—N5—C12—C7	84.0 (2)
N3—C5—C6—C7	−1.6 (2)	O11—N5—C12—C7	−94.7 (2)
O1—C1—C6—C5	175.27 (15)	O12—N5—C12—C11	−99.0 (2)
C2—C1—C6—C5	−5.1 (2)	O11—N5—C12—C11	82.3 (2)
O1—C1—C6—C7	−4.6 (2)		

### Hydrogen-bond geometry (Å, °)

**Table:**

*D*—H···*A*	*D*—H	H···*A*	*D*···*A*	*D*—H···*A*
C13—H13A···O3	0.96	2.45	2.926 (3)	111
C14—H14B···O10^i^	0.96	2.55	3.502 (3)	174
C14—H14C···O8^ii^	0.96	2.58	3.371 (3)	140

Symmetry codes: (i) *x*+1, *y*−1, *z*; (ii) −*x*+1, −*y*+2, −*z*+1.
